# Rapid Real-Time Polymerase Chain Reaction for *Salmonella* Serotyping Based on Novel Unique Gene Markers by Pangenome Analysis

**DOI:** 10.3389/fmicb.2021.750379

**Published:** 2021-09-21

**Authors:** Seung-Min Yang, Eiseul Kim, Dayoung Kim, Hyeon-Be Kim, Jiwon Baek, Seyoung Ko, Donghyuk Kim, Hyunjin Yoon, Hae-Yeong Kim

**Affiliations:** ^1^Institute of Life Sciences and Resources, Department of Food Science and Biotechnology, Kyung Hee University, Yongin, South Korea; ^2^Department of Molecular Science and Technology, Ajou University, Suwon, South Korea; ^3^School of Energy and Chemical Engineering, Ulsan National Institute of Science and Technology (UNIST), Ulsan, South Korea; ^4^School of Life Sciences, Ulsan National Institute of Science and Technology (UNIST), Ulsan, South Korea

**Keywords:** *Salmonella*, serotyping, pangenome analysis, detection, real-time PCR, gene marker

## Abstract

An accurate diagnostic method for *Salmonella* serovars is fundamental to preventing the spread of associated diseases. A diagnostic polymerase chain reaction (PCR)-based method has proven to be an effective tool for detecting pathogenic bacteria. However, the gene markers currently used in real-time PCR to detect *Salmonella* serovars have low specificity and are developed for only a few serovars. Therefore, in this study, we explored the novel unique gene markers for 60 serovars that share similar antigenic formulas and show high prevalence using pangenome analysis and developed a real-time PCR to detect them. Before exploring gene markers, the 535 *Salmonella* genomes were evaluated, and some genomes had serovars different from the designated serovar information. Based on these analyses, serovar-specific gene markers were explored. These markers were identified as genes present in all strains of target serovar genomes but absent in strains of other serovar genomes. Serovar-specific primer pairs were designed from the gene markers, and a real-time PCR method that can distinguish between 60 of the most common *Salmonella* serovars in a single 96-well plate assay was developed. As a result, real-time PCR showed 100% specificity for 199 *Salmonella* and 29 non-*Salmonella* strains. Subsequently, the method developed was applied successfully to both strains with identified serovars and an unknown strain, demonstrating that real-time PCR can accurately detect serovars of strains compared with traditional serotyping methods, such as antisera agglutination. Therefore, our method enables rapid and economical *Salmonella* serotyping compared with the traditional serotyping method.

## Introduction

The genus *Salmonella*, the causative agent of foodborne salmonellosis, can infect both animals and humans, leading to public health problems and economic loss (Gand M. et al., 2020a). Most *Salmonella* infections are caused by consuming contaminated water or food (Kasturi, 2020). Currently, *Salmonella* is divided into two species and six subspecies. Serotypes are further classified into more than 2,600 serovars following the White–Kauffman–Le Minor scheme, using antigenic agglutination reactions to three cell-surface antigens of somatic O, and flagellar H antigens denoted as H1 and H2 (Grimont and Weill, 2007; Yachison et al., 2017; Zhang et al., 2019; Gand M. et al., 2020a). As a reliable surveillance protocol is critical for detecting outbreaks or preventing their spread, using a differential serotyping method that identifies serogroups and serovars of *Salmonella* isolates from causative agents is important (Kasturi, 2020).

Traditional serotyping methods require numerous antisera, are labor-intensive, time-consuming, and complicated, and may produce ambiguous results (Hong et al., 2008; Xiong et al., 2017). Some isolates do not express antigens because of a single-nucleotide mutation in the genome, and some require multiple passes through semisolid media to enhance the flagella antigen expression (Ibrahim and Morin, 2018). As a result of these limitations, serotyping by antigenic agglutination and biochemical tests has been replaced by molecular serotyping (Zhang et al., 2019). For the rapid diagnosis or tracking of *Salmonella* serovars, epidemiological investigations have been conducted by molecular serotyping analysis based on pulsed-field gel electrophoresis, plasmid profiles, and polymerase chain reactions (PCRs) (Ozdemir and Acar, 2014; Gad et al., 2018; Yang et al., 2021). Notably, the PCR-based method is widely used for early diagnosis because it can diagnose a few bacteria in the specimen and can yield rapid results. However, as the PCR-based method for serotyping mainly uses markers within genes responsible for somatic and flagellar antigen expression, these genes do not detect strains that share the same antigenic formula. Thus, although PCR is reasonably rapid and inexpensive compared with conventional serotyping methods, the limiting factors of this assay are that molecular serotyping does not diagnose numerous serovars and focuses mainly on the most common serovars, such as *Salmonella* Typhimurium and Enteritidis (Ibrahim and Morin, 2018).

Previous studies have identified gene markers, such as *fimA*, *hilA*, *invA*, and *ttr*, that are useful in detecting *Salmonella* species (Laing et al., 2017; Gand M. et al., 2020b; Kreitlow et al., 2021). Moreover, for serovar identification, gene markers based on allelic variations in O and H antigen genes were used (Laing et al., 2017; Gand M. et al., 2020b). However, this gene marker cannot distinguish isolates that share the same antigenic formula. To overcome this problem, some studies have identified markers, such as STM0292, STM4200, STM4493, and STM2235 specific to Typhimurium and SEN1392 specific to Enteritidis, using comparative genomics (Akiba et al., 2011; Liu et al., 2012). These gene markers have specificity but have been screened using a limited number of genomes and cannot detect various serovars in a single reaction (Heymans et al., 2018; Ibrahim and Morin, 2018).

Advances in whole-genome sequencing technology have improved the understanding of the species and subtypes of pathogenic bacteria, and can provide information on the virulence of the underlying pathogenesis. Data obtained using whole-genome sequencing technology can be used to confirm paths of disease transmission and provide information on potential outbreaks (Ibrahim and Morin, 2018; Diep et al., 2019; Cooper et al., 2020). Therefore, whole-genome sequencing is currently used as a technique to obtain reliable and rapid serovar information. Recently, multiple *in silico* tools have been developed to determine *Salmonella* serovars from whole-genome sequence data (Zhang et al., 2015, 2019; Yoshida et al., 2016). SeqSero and Salmonella *In Silico* Typing Resource (SISTR), which can infer serovar predictions from analyses of somatic O and flagellar H antigens derived from whole-genome sequence data, are representative *in silico* analysis tools (Uelze et al., 2020). Whole-genome sequencing and *in silico* tools have many advantages in serotyping, for example, they reveal detailed genetic information on the characteristics of isolates and accurate serovar predictions, provided the database is sufficient (Ibrahim and Morin, 2018). However, whole-genome sequence-based method must be considered against costs associated with genome sequencing to analyze the many samples. This method also requires trained researchers with a specific skill set (Mellmann et al., 2017). To overcome the limitations of genome sequencing, primer sets for PCR targeting serovar-specific genes were recently developed. They show accuracy for serovar detection but still cannot detect a large number of serovars (Shang et al., 2021; Ye et al., 2021).

The purpose of this study is to evaluate *Salmonella* genomes by *in silico* serotyping, to select novel serovar-specific gene markers based on pangenome analysis, and to develop a real-time PCR method that can distinguish between 60 of the most common *Salmonella* serovars in a single 96-well plate by detecting unique serovar-specific gene markers.

## Materials and Methods

### *In silico* Serotyping

This study selected 60 serovars, which have been frequently isolated worldwide, are essential for public health, and are difficult to diagnose using traditional serotyping methods. The target serovars are as follows: Aberdeen, Agona, Albany, Anatum, Bareilly, Berta, Blockley, Braenderup, Brandenburg, Cerro, Choleraesuis, Corvallis, Derby, Dublin, Elisabethville, Enteritidis, Gallinarum, Give, Hadar, Heidelberg, I 4,[5],12:i:-, Infantis, Javiana, Kedougou, Kentucky, Kottbus, Litchfield, Livingstone, London, Manhattan, Mbandaka, Meleagridis, Menston, Minnesota, Mississippi, Montevideo, Muenchen, Muenster, Newington, Newport, Ohio, Oranienburg, Panama, Paratyphi B, Poona, Reading, Rissen, Saintpaul, Schwarzengrund, Senftenberg, Singapore, Stanley, Tennessee, Thompson, Typhi, Typhimurium, Uganda, Vinohrady, Virchow, and Weltevreden. The 535 *Salmonella* assembled genome sequences representing 60 serovars were obtained from the National Center for Biotechnology Information (NCBI) (Supplementary Table 1). All genomes used in this study were subjected to *in silico* serotyping using SeqSero2 version 1.2.1 and SISTR version 1.1.1. SeqSero used the assembled genome sequence as the input and the sequence was analyzed using Python code (Zhang et al., 2015, 2019). Also, serotyping and core-genome multilocus sequence typing (cgMLST) of whole-genome sequences were analyzed using a SISTR command-line tool.

### Pangenome Analysis and Discovery of Unique Gene Markers

The pangenome was analyzed using workflow for microbial pangenomic analysis with the Anvi’o package version 6.1 (Eren et al., 2015). Briefly, the assembled genome sequences were used as input and clustered based on the similarity of the amino acid sequences by the Markov cluster algorithm and NCBI’s blastp algorithm according to the developer recommendations (Kayansamruaj et al., 2019). Then the pangenome result was visualized using anvi-display-pan code of Anvi’o, and the genomes were organized based on the pan gene cluster frequencies.

The unique gene of each serovar was obtained using a Bacterial Pan Genome Analysis (BPGA) version 1.3 (Chaudhari et al., 2016). The annotated protein in fasta-format file was used as input, and the pangenome was analyzed with the default value (cut-off: 50%). All genomes were compiled into separate local databases, which include the core-genome composed of proteins common to genomes of the target serovar, and the pangenome is composed of entire proteins of all genomes. The unique genes of each serovar were gathered by comparing the pan and core-genome databases. The extracted unique genes were aligned with 65,815,883 sequences using the Basic Local Alignment Search Tool (BLAST). To verify the unique gene markers, the unique gene of each serovar was aligned with 535 *Salmonella* genomes using USEARCH version 9.0 (Edgar, 2010). Afterward, serovar-specific primer pairs were designed from selected gene markers. To increase the efficiency of primer pairs, the length was 18–30 base pairs, the guanine–cytosine (GC) content was designed to be 45–60%, and the melting temperature (Tm) value was 52–58°C (Abd-Elsalam, 2003). Representativeness of newly designed primer pairs were evaluated by *in silico* PCR. PCR was run from a web-based *in silico* PCR amplification^1^ software using 625 *Salmonella* genomes (Supplementary Table 2). The genomes used in the *in silico* PCR come from NCBI and EnteroBase.

### Cultured Bacterial Strains and DNA Extraction

The 199 *Salmonella* strains, 33 *Salmonella* species or *Salmonella enterica* strains, and 29 non-*Salmonella* strains used in this study are presented in Supplementary Table 3. All bacterial strains were grown in TSB at 37°C for 18 h under aerobic conditions. The cultured strains were collected by centrifugation at 13,600 × *g* for 5 min. Then, genomic DNA was extracted from the pellet using the DNeasy Blood & Tissue Kit (QIAGEN, Hilden, Germany), according to the manufacturer’s instructions. The concentration and purity of the genomic DNA extracted were measured using the MaestroNano^®^ spectrophotometer (Maestrogen, Las Vegas, NV, United States).

### Specificity and Accuracy for Developed Primer Pairs

Each 20-μl real-time PCR reaction mixture contained 500 nM of each primer pair, 10 μl of 2× Thunderbird SYBR^®^ qPCR Mix (Toyobo, Osaka, Japan), and 20 ng of template DNA. To avoid false-negative results, an internal standard targeting the bacterial 16S rRNA gene fragment was used (Aboutalebian et al., 2021). Amplification was conducted in a 7500 Real-Time PCR System (Applied Biosystems, Foster City, CA, United States), with an initial denaturation for 2 min at 95°C, followed by 30 cycles of 95°C for 5 s and 60°C for 30 s. The melting curve was generated according to the following conditions: 95°C for 15 s, 60°C for 1 min, 95°C for 30 s, and 60°C for 15 s. The specificity of the primer pairs developed was tested against each *Salmonella* strain as well as each non-*Salmonella* reference strain. Primer pairs cross reactivity across all of the serovars were evaluated. To evaluate the accuracy, genomic DNA for each serovar were serially diluted, and then standard curves were generated in triplicate using diluted DNA from 0.0002 to 20 ng. The results obtained were analyzed using 7500 software version 2.3 (Applied Biosystems).

### Evaluation of Real-Time Polymerase Chain Reaction With a Single 96-Well Plate

A real-time PCR method was developed that can distinguish between 60 of the most common *Salmonella* serovars in a single 96-well plate. The real-time PCR was designed so that each primer pair was run independently in a single 96-well plate (Supplementary Figure 1). Each well contained different primer pair, and the genomic DNA from a single isolate was added to each well. The serovar of strain was determined as serovar corresponding to the primer pair included in the well in which amplification occurred. The real-time PCR developed in this study was evaluated using 189 *Salmonella* strains whose serovars were confirmed through antisera agglutination and 33 strains whose serovars were unknown. Each strain was tested against each of the primer pairs. For serovar diagnosis of isolates, genomic DNA of the isolate was added to each well of the reaction plate containing different primer pair and 2× Thunderbird SYBR^®^ qPCR Mix (Toyobo). Then, real-time PCR was conducted in the 7500 Real-Time PCR System (Applied Biosystems). The conditions were the same as those described in the previous “Specificity and Accuracy for Developed Primer Pairs” section.

### Traditional Serotyping Using Antisera Agglutination

The antigenic formulas of strains were determined by the World Health Organization Collaborating Center for Reference and Research on *Salmonella*, located at the Pasteur Institute, and the serovar name was assigned by the White–Kauffmann–Le Minor scheme (Grimont and Weill, 2007). All strains were cultured in brain heart infusion (BD Difco, Sparks, MD, United States) and motility GI medium (BD Difco) to determine somatic and flagellar antigens. The somatic antigen was confirmed by the slide agglutination reaction using antisera (BD Difco). The flagellar phase was activated by the motility test and fixed by treatment with 0.6% formalin. The flagellar antigen was determined by an aggregation reaction in glass test tubes by mixing with an antisera solution (BD Difco). The serotyping results of antisera agglutination were compared with the results of the real-time PCR method.

## Results and Discussion

### *In silico* Serotyping

Whole-genome sequencing data have been used for serotype diagnosis or subtyping of *Salmonella* (Zhang et al., 2019; Uelze et al., 2020). SeqSero is a web-based serotyping tool that can predict many *Salmonella* serovars using whole-genome sequence data based on a database of *Salmonella* serovar determinants (Ibrahim and Morin, 2018). This tool extracts the relevant genomic regions of cell-surface antigens, such as the *rfb* gene cluster to determine the O antigen and the *fliC* and *fljB* genes to determine H1 and H2 antigens, from the genome assemblies or raw sequencing reads and aligns these genes to the curated database using BLAST (Zhang et al., 2015; Ibrahim and Morin, 2018). This software then determines the serovar according to the White–Kauffmann–Le Minor scheme.

In this study, 511 genomes (95.51%) were predicted as correct serovars by SeqSero, and the remaining 24 genomes (4.49%) were predicted as incorrect serovars (Supplementary Table 4). Of the 24 genomes that produced incorrect serovars, eight genomes were predicted to be serotypes inconsistent with serovar nomenclature reported to NCBI, and 13 genomes indicated two or more serotypes (Table 1). This shows that as the serotype by SeqSero is determined only by the O and H antigens, these genomes show that two or more serovars were indicated for similar serovars, such as Gallinarum or Enteritidis, Paratyphi C or Choleraesuis or Typhisuis, and Albany or Duesseldorf. Additionally, some genomes (*n* = 4) were determined to be partial serovars lacking O or H1 or H2-antigens, and accurate serovar information could not be extracted. The reason for this appears to be the failure to extract the serovar determinant from the assembled genomes (Zhang et al., 2015).

**TABLE 1:** Genomes predicted to incorrect serovar among 535 *Salmonella* genomes by *in silico* serotyping.

Serovars	Predicted serovar by SeqSero2	Predicted serovar by SISTR
Albany ATCC 51960	Albany or Duesseldorf (8:z4,z24:-)	Albany
Albany sg_wt5	Albany or Duesseldorf (8:z4,z24:-)	Albany
Albany CFSAN103854	Albany or Duesseldorf (8:z4,z24:-)	Albany
Albany R17.5974	Albany or Duesseldorf (8:z4,z24:-)	Albany
Albany R16.0556	Albany or Duesseldorf (8:z4,z24:-)	Albany
Choleraesuis SC-B67	Paratyphi C or Choleraesuis or Typhisuis (7:C:1,5)	Choleraesuis
Choleraesuis A50	Paratyphi C or Choleraesuis or Typhisuis (7:C:1,5)	Choleraesuis
Choleraesuis ATCC 10708	Paratyphi C or Choleraesuis or Typhisuis (7:C:1,5)	Choleraesuis
Choleraesuis C500	Paratyphi C or Choleraesuis or Typhisuis (7:C:1,5)	Choleraesuis
Derby 2013LSAL02374	I -:f,g:- (-:f,g:-)	Derby
Enteritidis 92-0392	Typhimurium (4:i:1,2)	Typhimurium
Gallinarum ATCC 9120	Gallinarum or Enteritidis (9:g,m:-)	Gallinarum
Gallinarum 13036	Gallinarum or Enteritidis (9:g,m:-)	Gallinarum
Gallinarum 19945	Gallinarum or Enteritidis (9:g,m:-)	Gallinarum
Gallinarum S4037-07	Gallinarum or Enteritidis (9:g,m:-)	Gallinarum
Give S5-487	Give (3,10:l,v:1,7)	E1:l,v:l,z13,z28
London CFSAN001081	-:l,v:1,6 (-:l,v:1,6)	-:e,n,x,z15:1,6
Mbandaka CFSAN076213	-:z10:e,n,z15 (-:z10:e,n,z15)	Mbandaka
Mississippi SAL-19-VL-SD-NC-0011	I -:b:1,5 (-:b:1,5)	Mississippi
Newington 261358	Anatum (3,10:e,h:1,6)	Anatum
Newington 95006	Anatum (3,10:e,h:1,6)	Anatum
Paratyphi B SARA61	Agona (4:f,g,s:-)	Agona
Typhimurium TW-Stm6	I 4,[5],12:i:- (4:i:-)	I 4,[5],12:i:-
Typhimurium FORC50	Enteritidis (9:g,m:-)	Enteritidis
Typhimurium FORC098	Tennessee (7:z29:-)	Tennessee

Salmonella *In Silico* Typing Resource also determines the serovar according to the White–Kauffmann–Le Minor scheme, based on their databases of *Salmonella* serotype determinants (*wzx*/*wzy*, *fliC*, and *fljB* alleles) (Yoshida et al., 2016). To resolve the ambiguous serovar designations resulting from antigen determination, SISTR uses the novel 330 locus cgMLST analysis, and together, these two determinants are used to provide an overall serovar prediction (Yoshida et al., 2016). As a result of SISTR analysis, 526 genomes (98.32%) were predicted as correct serovars (Supplementary Table 5), whereas nine genomes (1.68%) were predicted as incorrect serovars (Table 1).

*In silico* serotyping analyses of the genomes obtained from the NCBI showed 95.51 and 98.32% accuracy on SeqSero and SISTR platforms, respectively. These data are consistent with the success rates of each platform reported in previous studies (Zhang et al., 2015; Yoshida et al., 2016). However, some limitations of whole-genome sequencing also exist. Previous studies have reported that SeqSero provided two possible serovars that share the same antigenic formula but differed in the minor O antigen factor (Ibrahim and Morin, 2018). In this study, serovars such as Anatum (3,10:e,h:1,6) and Newington (Anatum var. 15^+^, 3,10,15:e,h:1,6) shared similar antigenic formulas, yielding only Anatum serovars. Moreover, *in silico* serotyping tools could not predict some genomes. These unpredicted serovars may infrequently be separated, or possibly there were some gaps in their databases (Ibrahim and Morin, 2018).

### Genome Evaluation

Previous studies have reported that the NCBI has often misclassified genomes, so the genome evaluation should be conducted before using the genome obtained from the NCBI (Kim et al., 2020, 2021). Therefore, the genomes used in this study were evaluated to prevent incorrect results obtained in selecting unique serovar genes.

**FIGURE 1:**
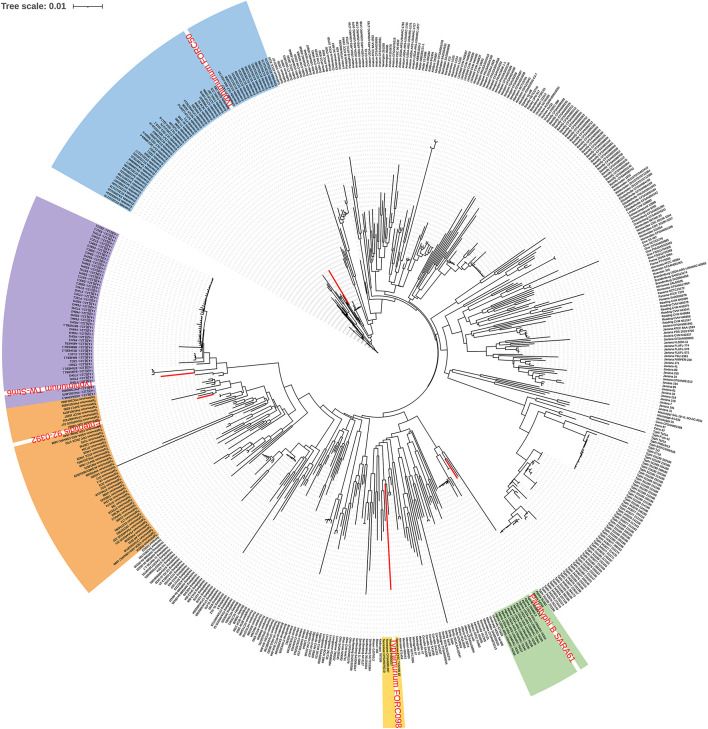
Pangenome distribution of the 535 *Salmonella* genomes. The red letters in the circular dendrogram indicates the strains predicted by the incorrect serovars. The orange, purple, blue, green, and yellow backgrounds represent Typhimurium, I 4,[5],12:i:-, Enteritidis, Agona, and Tennessee, respectively.

As a result of phylogeny based on the pangene cluster frequencies among the 535 genomes, most genomes were clustered according to serovar types (Figure 1). Serovars with similar antigenic formula types, such as Typhimurium and I 4,[5],12:i:-, were adjacent but clustered into different groups. These two serovars differ by only one flagellar antigen (1,4,[5],12:i:1,2 vs. 4,[5],12:i:-) in their antigenic formulas. However, some Enteritidis, Typhimurium, and Paratyphi B strains were clustered with different serovars. Paratyphi B SARA61 was clustered into Agona; Typhimurium TW-Stm6, FORC50, and FORC098 were clustered into I 4,[5],12:i:-, Enteritidis, and Tennessee, respectively; Enteritidis 92-0392 was clustered into Typhimurium. Most genomes were clustered with the same serovar group because of *in silico* serotyping analyses. This is consistent with a previous study that reported that similar or rare serotypes could produce unpredicted serovar results using the SeqSero database (Ibrahim and Morin, 2018).

### Identification of Serovar-Specific Gene Markers

A total of 2,440,535 genes yielded a pangenome size of 15,853 genes. The core genome comprises 1,215 genes; the accessory genome, 11,156 genes; and the unique genome, 3,482 genes. The core genomes for each serovar included from 3,065 to 4,702 genes, and unique genomes contained from 1 to 160 genes. The unique genes considered specific for each serovar were identified by BLAST analysis, and genes rarely present in other bacteria and specific to the serovar were selected. Further, a unique gene marker of each serovar was finally selected considering the GC content and sequence length suitable for the primer design. The 60 gene markers selected by the analysis were confirmed to be genes present in all target serovars and absent in other *Salmonella* serovars. Information on these gene markers is shown in Table 2.

**TABLE 2:** Information of gene markers obtained pangenome analysis.

Serovar	Protein name	Accession no.	Size (bp)
Aberdeen	Glycosyl transferase family 1	SQH80039.1	1,101
Agona	Hypothetical protein	ACH51233.1	1,284
Albany	Type II restriction endonuclease subunit R	APV71773.1	1,404
Anatum	Hypothetical protein	AHW13895.1	681
Bareilly	Hypothetical protein	QGJ28052.1	1,113
Berta	Hypothetical protein	ESH53086.1	522
Blockley	RNA-dependent DNA polymerase	EBW8522522.1	1,193
Braenderup	Hypothetical protein	ASO31941.1	384
Brandenburg	Restriction endonuclease subunit S	KNN28838.1	642
Cerro	Hypothetical protein	ALI12694.1	1,008
Choleraesuis	LD-carboxypeptidase A	EFZ05851.1	966
Corvallis	Hypothetical protein	AWD06828.1	990
Derby	Replicase family protein	KMM40244.1	846
Dublin	Conserved hypothetical protein	ACH74104.1	1,161
Elisabethville	Hypothetical protein	EBS4171315.1	1,116
Enteritidis	Putative phage membrane protein	CAR32961.1	537
Gallinarum	Hypothetical protein	AKW12500.1	1,020
Give	DUF1269 domain-containing protein	OZU63064.1	1,662
Hadar	Hypothetical protein	KKD96963.1	507
Heidelberg	Abortive infection protein	QGF81602.1	1,722
I 4,[5],12:i:-	Hypothetical protein	QGX33576.1	231
Infantis	USG protein	CEI43307.1	873
Javiana	Hypothetical protein	QDI87568.1	714
Kedougou	FRG domain-containing protein	EBU9135186.1	1,410
Kentucky	Hypothetical protein	ASO54323.1	1,488
Kottbus	Phage tail protein	EDL0140664.1	1,479
Litchfield	Hypothetical protein	KNL82901.1	1,110
Livingstone	ABC transporter permease subunit	HAB5790955.1	909
London	Hypothetical protein	ESJ48773.1	536
Manhattan	Reverse transcriptase	ASO47481.1	2,019
Mbandaka	Hypothetical protein	AYP83194.1	1,824
Meleagridis	Hypothetical protein	TSE72805.1	2,961
Menston	DUF4238 domain-containing protein	ECG3796773.1	918
Minnesota	Amylovoran biosynthesis protein AmsE	APV92647.1	810
Mississippi	AAA family ATPas	EDN5268668.1	4,248
Montevideo	Hypothetical protein	AHW10654.1	1,767
Muenchen	Hypothetical protein	QGH07522.1	810
Muenster	Hypothetical protein	AUM47824.1	1,365
Newington	Hypothetical protein	ECJ7339255.1	195
Newport	Hypothetical protein	ALP98662.1	843
Ohio	Adenosylhomocysteinase	AXE13015.1	1,152
Oranienburg	Hypothetical protein	AUM45051.1	1,392
Panama	Hypothetical protein	AKW08656.1	1,194
Paratyphi B	Putative bacteriophage protein	ESF91711.1	1,200
Poona	Glycosyltransferase	SQJ10189.1	1,017
Reading	Hypothetical protein	KNL67516.1	681
Rissen	Helicase domain-containing protein	ELX22432.1	2,502
Saintpaul	Hypothetical protein	ASO37389.1	2,277
Schwarzengrund	Y4bN protein	ACF91479.1	2,445
Senftenberg	FRG domain	CRY85796.1	822
Singapore	DNA adenine methylase	EDA1367050.1	673
Stanley	Hypothetical protein	QBG28938.1	2,142
Tennessee	Hypothetical protein	AMW53127.1	306
Thompson	Hypothetical protein	AGX13681.1	1,779
Typhi	Host-nuclease inhibitor protein Gam	ALG16175.1	531
Typhimurium	Putative outer membrane	AAL23519.1	558
Uganda	BREX-1 system phosphatase PglZ type A	TSB75053.1	2,604
Vinohrady	Haloacid dehalogenase-like hydrolase	ECE8801113.1	684
Virchow	Hypothetical protein	ESE99108.1	849
Weltevreden	Putative phosphatase	CUS01310.1	648

**FIGURE 2:**
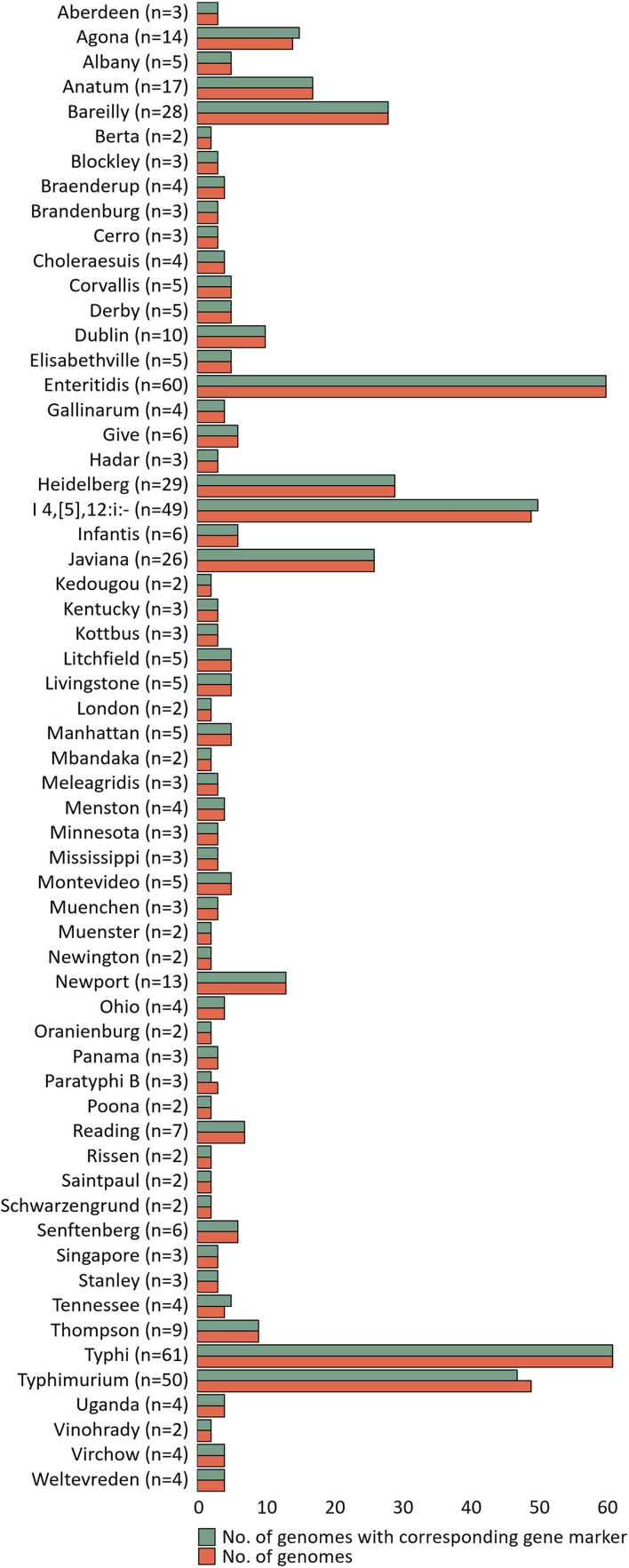
Evaluation of specificity unique gene markers. The figure shows the presence or absence of unique gene markers in 535 genomes. The number of analyzed genomes for each serovar is shown in the green bar, and the number of genomes with gene marker corresponding to that serovar is shown in the red bar.

The specificity of gene markers was evaluated using 535 genomes by *in silico* analysis. As a result, gene markers were present in the genomes of most target serovars (Figure 2). Notably, most of the gene markers shared 99–100% of the sequence identified in the target serovars, and 0–50% of the sequence identified against other serovars. In contrast, for the serovar Enteritidis, 60 genomes showed 99–100% identity, but in one genome, the Enteritidis gene marker was not found. Among them, Enteritidis 92-0392 contained the Typhimurium gene marker (100% identity) instead of the Enteritidis gene marker, and this genome was determined as Typhimurium in the pangenome analysis and *in silico* serotyping. For the Typhimurium gene marker, 46 genomes showed 99–100% identity, but in three genomes, the Typhimurium-specific gene marker could not be found. Typhimurium TW-Stm6, FORC50, and FORC098 showed 100% identity to I 4,[5],12:i:-, Enteritidis, and Tennessee-specific gene markers instead of the Typhimurium-specific gene marker, respectively. For serovar Paratyphi B, two genomes showed 100% identity, but in one genome, the Paratyphi B-specific gene marker could not be found. Paratyphi B SARA61 showed 100% identity to Agona gene markers instead of the Paratyphi B gene marker. As mentioned above, misclassified genomes were reclassified. Therefore, Agona, I 4,[5],12:i:-, and Tennessee had more genomes with corresponding gene markers than the number of genomes analyzed (Figure 2). In contrast, misclassified genome absent the corresponding gene markers, so some serovar had more analyzed genomes than the number of corresponding gene markers. Based on the pangenome analysis, serovar-specific primer pairs that can accurately detect *Salmonella* serovars were developed (Table 3). An Infantis-specific primer pair was developed in the previous study (Yang et al., 2021).

**TABLE 3:** Sequences of serovar-specific primer pairs for real-time PCR.

Serovar	Name	Primer sequences (5′→3′)	Size (bp)
Aberdeen	Aberdeen-F	AAC AAC GGG TAC AGG GAT TA	131
	Aberdeen-R	AAT CCT TAT TAT CGT CCC CA	
Agona	Agona-F	GCA TCT GGC GGT AAG TCA TA	190
	Agona-R	GTG AGC GTA ATG GGG ATG TA	
Albany	Albany-F	TAG TCA GGT AGC ACC GAG TT	163
	Albany-R	ACG CCA TGT AGA TTC GTT AT	
Anatum	Anatum-F	AAA GCA CCC TGA GTC AGA TG	143
	Anatum-R	CAA GTC CAC CGA CTG CTC TA	
Bareilly	Bareilly-F	GGT GGT AGT ACC AAG GAT GT	114
	Bareilly-R	ACT GCT AGT TCC TCC GTA AG	
Berta	Berta-F	TAA CCG AGG AGC CAA CAG TG	145
	Berta-R	CGG AGA GGG TCC AGT TGT TT	
Blockley	Blockley-F	TCC GTG GTT CAT GAG CAG TT	124
	Blockley-R	GAA GGT CAT CAC GCC TAG GT	
Braenderup	Braenderup-F	GGA GAA TGC TTG CAG GAA GA	156
	Braenderup-R	GCT GGT TCA AAG TAA TGC GG	
Brandenburg	Brandenburg-F	TGG TTC TAC ACC TAA AGG TGG	108
	Brandenburg-R	TAC GCG ACA TCA TCT AGC CT	
Cerro	Cerro-F	CGT TTC TCC GTT TAT GTG GA	157
	Cerro-R	GGC ATT GTT ACA GAC AAA GC	
Choleraesuis	Choleraesuis-F	GCT CCA TCT TCG CCA ATT GA	125
	Choleraesuis-R	TCC AGT AAC GCT GTA GGC TCT	
Corvallis	Corvallis-F	AAG CGT TTA TTG GAG GCT GA	139
	Corvallis-R	CGC TGT TCG ATG CTT CAA GT	
Derby	Derby-F	TGC GTC CGT TGT TCA ATG TG	106
	Derby-R	CGT TGT ACG CCA TTC AGC TT	
Dublin	Dublin-F	GCG TCA AGG TTT ATT GAA TCG	114
	Dublin-R	GGA TGT CAA TCG CTG TTG TC	
Elisabethville	Elisabethville-F	GAC CAC GAC CGG TAC AGC AA	178
	Elisabethville-R	CGG CGG ATA CTG CAC ACG AA	
Enteritidis	Enteritidis-F	TTG GTA AAT CCG TCG GAC AA	105
	Enteritidis-R	AAT CGC TAC GCG CCT CAA TA	
Gallinarum	Gallinarum-F	CGA CGG TCG TCA ATC CTA CT	113
	Gallinarum-R	ATC AAC CAC AGC CGT AGC AG	
Give	Give-F	TCA TTG GCA CTG GTG AGT CG	103
	Give-R	CCT TCA ATG CCT GGC ACA TC	
Hadar	Hadar-F	GTG AGT CTT TTT CGG TGA TA	162
	Hadar-R	ATC TCA CCC ATT CAC AGA TA	
Heidelberg	Heidelberg-F	CGG CGA ATT AAT CAT AAG CG	105
	Heidelberg-R	CTC TCA CCT GAT TTT GCC TGT	
I 4,[5],12:i:-	I 4,[5],12:i:-F	AAG TGC GCC AGT TAG CTT CT	113
	I 4,[5],12:i:-R	GGT ATC GCC GTC AAT ACA CA	
Infantis	Infantis-F	GGT CGA GAT GGG TAT GTA GC	109
	Infantis-R	CAG GAG TTC CTG CGC AAC CA	
Javiana	Javiana-F	TGG CTA CTC AGG CAG TAC TA	112
	Javiana-R	AGC ATA ACT CCG TGA GTT TT	
Kedougou	Kedougou-F	GTC AGC CTT CCT GAA GTC AT	102
	Kedougou-R	CTC GCG TTC ATA CAA TCC TG	
Kentucky	Kentucky-F	CGA TAA CTT GCG GAG TGA CA	141
	Kentucky-R	TTC CAC CGT TGG CCT CAT AA	
Kottbus	Kottbus-F	GCG TCT GAC TGG AGC AGA TT	108
	Kottbus-R	ACC ACA GTC AAC GCC TAG GT	
Litchfield	Litchfield-F	CAG ACT TAA TAG AGG ACC CA	143
	Litchfield-R	CTC CGT TTC ATT CCA TCC AC	
Livingstone	Livingstone-F	TCT GCG CAC AGG CGA ATT CT	103
	Livingstone-R	CAG ACG CTT AGA GAC GGT GTG A	
London	London-F	GGC TCA TCC GGA ACG AAC AA	141
	London-R	CAA GCG AGC TTA TAG GCG TAG	
Manhattan	Manhattan-F	GCT GAT GCA GCG TAG CAA TA	128
	Manhattan-R	GCT CAC TAA GAA GGC ATG ACT C	
Mbandaka	Mbandaka-F	ATC GAG GAT CCA AGC ATC AG	130
	Mbandaka-R	GGA AAA CAC CAA GGA CTT CG	
Meleagridis	Meleagridis-F	TGG CGA TAT ACC GGT TAC CT	118
	Meleagridis-R	TCC GCG TAA CTG ATC ACT TC	
Menston	Menston-F	TAG TGT TGC GAC GGA GCT AA	101
	Menston-R	TTC GAA CAG CCA GCA GTG AA	
Minnesota	Minnesota-F	GCG GCT ACA AGC ATC ATC AT	117
	Minnesota-R	CCT TCC CAA CTC GAA CTT TAA C	
Mississippi	Mississippi-F	CCA CGA CAC CAT CAA TCA TC	115
	Mississippi-R	GCA ATA GGC GGT ACT AAG GA	
Montevideo	Montevideo-F	CCA ACC TGG CCA ACA AGA TT	120
	Montevideo-R	GAA CTG TCG CAC ACC GAT TC	
Muenchen	Muenchen-F	GCA CGT ATG CAG ATC GAA GA	134
	Muenchen-R	GTT AGC CGT TCC ACT GAC AA	
Muenster	Muenster–F	CAC CTC CTG AGA CTG AAG AA	175
	Muenster–R	CCG TCA TTT AGA TAA GGA AG	
Newington	Newington-F	CGT AGT CGT GGT TGC TGG TA	121
	Newington-R	TGC AGC AAG TAT GAC GAA TG	
Newport	Newport-F	GTT GCC AAA AAG CAC AAT GA	117
	Newport-R	AGC TCG AGT AAT CCG CAT GA	
Ohio	Ohio-F	CGA TAA TTG CCG CCT TCT GA	119
	Ohio-R	TCA GCA GGA GCG TGA CAG TT	
Oranienburg	Oranienburg-F	GCT GAG ATT GTG ATT CCA CC	101
	Oranienburg-R	CGC TGT TCT AACCTT GAG GA	
Panama	Panama-F	GCT CAA TTA GAT CCA ACA GC	104
	Panama-R	GAC TGG AGT GCA AGG TAG TT	
Paratyphi B	Paratyphi B-F	CGA TGG CTC GAT CCT GTT CAA G	120
	Paratyphi B-R	GTC CGG CGG ACA ACT ATC AAC C	
Poona	Poona-F	TGT TGG AGG ATG CCA TGA GT	127
	Poona-R	AAG GAC AGC TTG CGT ATG GA	
Reading	Reading-F	GCG AAT GGC GAT AAG GTT GA	128
	Reading-R	GCT CCG ATC AAA ACA TGA GTC	
Rissen	Rissen-F	GAG CTA GTT GCC GAA TCG AA	117
	Rissen-R	CCG AAT GAA TGC TGG CAA GT	
Saintpaul	Saintpaul-F	TGA TGG GAT ATC TCG CAA CA	154
	Saintpaul-R	GCC GCT ATG GAA CTT ATT CG	
Schwarzen- grund	Schwarzen- grund-F	TGG CGC TAT TAC CAC TGA TG	131
	Schwarzen- grund-R	CAA TTG CTG CGG ACC AAC TA	
Senftenberg	Senftenberg-F	GAG CAG GAT ATG CGC GAC TA	131
	Senftenberg-R	GTT GCT GCT CCA TGG TGT TG	
Singapore	Singapore-F	CCA GCA GAT AGG AAC ATA GG	109
	Singapore-R	GGA ACG ATA AGG CAT CAT CA	
Stanley	Stanley-F	TAC TGG CCT GGT GTC TTG TT	120
	Stanley-R	GTA TCC ATT GCC AGC GAG TA	
Tennessee	Tennessee-F	ACA AAC AAG CCT TCA GGT GG	102
	Tennessee-R	CAG CTC CTT CTG TTG CTC AT	
Thompson	Thompson-F	TAT TGC AAC AAT GAG GCC CTC T	126
	Thompson-R	GTC GCG ATT CTG AAC CGT GTC	
Typhi	Typhi-F	TGA GGC ACA CCG TGA TGA ACT G	118
	Typhi-R	ATT ATC CGC TCC GCG AAT GCT	
Typhimurium	Typhimurium-F	CGG CGG TAA TAC GAT GAA CT	126
	Typhimurium-R	CTT GCT GTC AGT GCT GTC TT	
Uganda	Uganda-F	AAG ATG TCT GGC ATG GGC AA	106
	Uganda-R	CGG GCT CCA CCA ACA AAA TG	
Vinohrady	Vinohrady-F	GAA GGA TTG CGA TTC GCT TT	117
	Vinohrady-R	GGA TGA CAT CAG CGA GTT CT	
Virchow	Virchow-F	GCC ACT GAT GAG ATG GAG TA	172
	Virchow-R	ACT CGC CAT CAG CAA TAC AC	
Weltevreden	Weltevreden-F	AAC CGG ATC CTG AGC CAT AC	111
	Weltevreden-R	CCG CTG CAA TAG CTG ATC TT	
Internal standard^1^	U bacteria-F	ATG TTG GGT TAA GTC CCG	265–267
	U bacteria-R	CTA GCG ATT CCR RCT TCA	

*^1^Internal standard primer pair was developed by Aboutalebian et al. (2021).*

In this study, our method was applied against 232 *Salmonella* strains. However, since the number of strains included in some serovars (e.g., Aberdeen, Berta, Cerro, Hadar, Kedougou, etc.) is rarely isolated, only a small number of strains were analyzed. *In silico* PCR was performed using the 625 genomes to determine whether marker genes are representative of each serovar and whether the accuracy can maintain high enough once it is applied to more strains of these serovars. All primer pairs were successfully amplified for corresponding genome sequences (Supplementary Table 2). Therefore, *in silico* PCR results revealed that the marker gene was representative for each serovar.

### Specificity and Accuracy of the Developed Real-Time Polymerase Chain Reaction

Pangenome analysis based on the whole-genome sequence can efficiently select serovar-specific gene markers using large-scale genomes. The classification of pathogenic bacteria to their correct taxonomy using whole-genome sequencing shows reproducibility and accuracy, but is expensive and requires additional bioinformatics analysis (Ibrahim and Morin, 2018). In contrast, the PCR-based method can rapidly detect pathogenic bacteria with high accuracy and sensitivity (Abubakar et al., 2007; Chen et al., 2010). This method can cost-effectively detect many isolates with relatively simple procedures; it also has potent sensitivity and specificity (Hoorfar, 2011; Xiong et al., 2018). It is crucial to develop the primer pair with high specificity as the accuracy of these PCR-based assays depends mainly on the specific gene or primer pair. Therefore, in this study, a real-time PCR method was developed for serotyping by detecting unique gene markers obtained through pangenome analysis.

**FIGURE 3:**
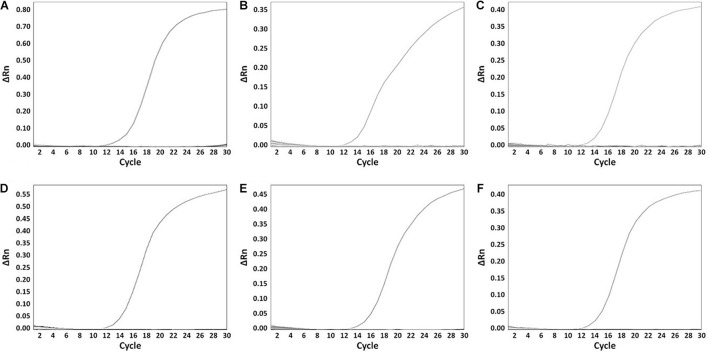
The specificity of serovar-specific primers. **(A)** Specificity of Bareilly primer pair, amplification curve: Bareilly MFDS 1007637; **(B)** Specificity of Enteritidis primer pair, amplification curve: Enteritidis MFDS 1010897; **(C)** I 4,[5],12:i:- primer pair, amplification curve: MFDS 1004858; **(D)** Specificity of Montevideo specific primer pair, amplification curve: CCARM 8189; **(E)** Specificity of Typhi primer pair, amplification curve: ATCC 33459; **(F)** Specificity of Typhimurium primer pair, amplification curve: ATCC 19585. ΔRn value means Rn (fluorescent signal from SYBR Green) value of an experimental response minus the Rn value of the baseline signal.

**FIGURE 4:**
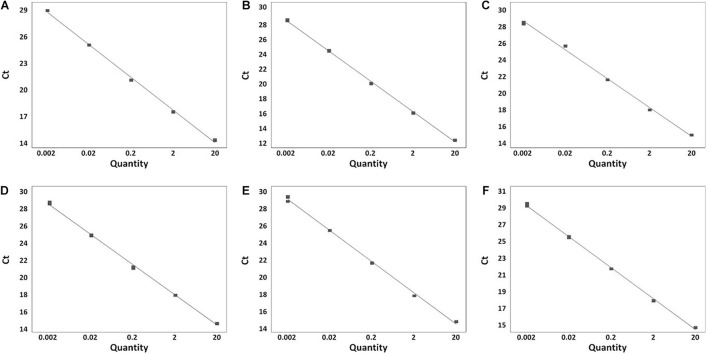
Real-time PCR standard curve. **(A)** Bareilly MFDS 1007637 standard curve (*y* = –3.58 x + 29.779, *R*^2^ = 0.998); **(B)** Enteritidis MFDS 1010897 standard curve (y = –3.39 x + 19.106, *R*^2^ = 0.999); **(C)** I 4,[5],12:i:- MFDS 1004858 standard curve (*y* = –3.66 x + 18.591, *R*^2^ = 0.997); **(D)** Montevideo CCARM 8189 standard curve (*y* = –3.45 x + 19.009, *R*^2^ = 0.999); **(E)** Typhi ATCC 33459 standard curve (*y* = –3.67 x + 21.768, *R*^2^ = 0.999); **(F)** Typhimurium ATCC 19585 standard curve (*y* = –3.61 x + 19.372, *R*^2^ = 0.999).

A total of 199 *Salmonella* strains and 29 non-*Salmonella* strains were used to develop a real-time PCR method that is specific and accurate. Amplification plots for the most frequently isolated six serovars worldwide are shown in Figure 3, and the result on the remaining serovars is in Supplementary Table 6. The genomic DNA across serovars yielded a detectable amplicon for the target primer pair, whereas those from all non-target *Salmonella* did not generate any signal (Figure 3). The Ct value ranges were 11.49–18.07 for each *Salmonella* serovar strain (Supplementary Table 6). Thus, primer pairs developed in this study were considered specific for the identification and detection of individual *Salmonella* serovars. Serial dilution was used on the genomic DNA of *Salmonella* reference strains to confirm the accuracy of the real-time PCR assay. All *Salmonella* serovar-specific primer pairs showed a linear relationship over the range of 0.002–20 ng. The slopes for the primer pairs of Bareilly, Enteritidis, I 4,[5],12:i:-, Montevideo, Typhi, and Typhimurium were −3.589, −3.395, −3.66, −3.457, −3.677, and −3.61, respectively, and the *R*^2^ values were ≥0.997 (Figure 4). The primer pairs for the remaining 54 serovars also showed that slope values were −3.19 to −3.683, and the *R*^2^ values were ≥0.996 (Supplementary Table 7). To show high efficiency, the slope value should be −3.1 to −3.9, and the *R*^2^ value ≥ 0.996 for the standard curve (Broeders et al., 2014). The slope and *R*^2^ values of all primer pairs developed in this study were within these ranges.

### Evaluation of Real-Time Polymerase Chain Reaction and Validation by Traditional Serotyping

The real-time PCR method developed in this study was evaluated in a single 96-well plate using 222 *Salmonella* strains. Moreover, serovars of some strains were identified using the antisera agglutination method and compared with real-time PCR results. Real-time PCR competency was checked using an internal standard. Of 222 strains, 189 strains are known at the serovar level, and the remaining 33 were strains with unknown serovars. The real-time PCR result determined that the corresponding serovars were detected when amplified in a well containing a serovar-specific primer pair. All strains were amplified in wells containing a specific primer pair and internal standard primer pair, whereas no amplification was observed in the other wells (Supplementary Table 8). The internal standard primer pair was amplified in 222 *Salmonella* strains and 29 non-*Salmonella*, and the Ct value ranged from 10.03 to 14.95. Serovars of all strains were identified accurately by real-time PCR, and the result was identical to the serotyping result of the antisera agglutination method (Table 4). Anatum and Newington presented similar antigenic formulas as the result of antisera agglutination, but two serovars were distinguishable in real-time PCR. To verify our real-time PCR method, serovars were determined for 33 isolates identified down to the genus or species level obtained from the Korea Veterinary Culture Collection (KVCC) (Table 5). As a result, 33 isolates were determined as 13 different serovars, such as I 4,[5],12:i:-, Enteritidis, Blockley, Hadar, Livingstone, Mbandaka, Montevideo, Rissen, Typhimurium, Infantis, Virchow, London, and Senftenberg. Therefore, our results suggest that the 60 primer pairs and real-time PCR method developed in this study are 100% accurate in detecting *Salmonella* serovars. However, in this study, few strains were used in some serovars that may influence the identification of the real marker genes and detecting accuracy.

**TABLE 4:** Comparison of serotyping results through antisera aggregation and real-time PCR.

Serovar (no. of strains)	Antigenic formulas^1^	No. of amplified strains^2^
Aberdeen (*n* = 2)	11:i:1,2	2
Agona (*n* = 4)	1,4,[5],12:f,g,s:[1,2]	4
Albany (*n* = 1)	8,20:z_4_:z_23_:-	1
Anatum (*n* = 2)	3,{10}{15}{15,34}:e,h:1,6	2
Bareilly (*n* = 8)	6,7,14:y:1,5	8
Berta (*n* = 1)	1,9,12:[f],g,[t]:-	1
Blockley (*n* = 2)	8:k:1,5	2
Braenderup (*n* = 4)	6,7,14:e,h:e,n,z_15_	4
Brandenburg (*n* = 2)	4,[5],12:l,v:e,n,z_15_	2
Cerro (*n* = 2)	6,14,18:z_4_,z_23_:[1,5]	2
Choleraesuis (*n* = 1)	7:c:1,5	1
Corvallis (*n* = 1)	8,20:z_4_,z_23_:[z6]	1
Derby (*n* = 5)	1,4,[5],12:f,g:[1,2]	5
Dublin (*n* = 2)	1,9,12:g,p:-	2
Elisabethville (*n* = 1)	3,{10}{15}:r:1,7	1
Enteritidis (*n* = 21)	1,9,12:g,m:-	21
Gallinarum (*n* = 1)	1,9,12:-:-	1
Give (*n* = 2)	3,{10}{15}{15,34}:l,v:1,7	2
Hadar (*n* = 7)	8:z10:e,n,x	7
Heidelberg (*n* = 4)	1,4,[5],12:r:1,2	4
I 4,[5],12:i:- (*n* = 2)	4,[5],12:i:-	2
Infantis (*n* = 8)	6,7,14:r:1,5	8
Javiana (*n* = 1)	1,9,12:l,z_28_:1,5	1
Kedougou (*n* = 2)	1,13,23:i:l,w	2
Kentucky (*n* = 2)	8,20:i:z_6_	2
Kottbus (*n* = 1)	8:e,h:1,5	1
Litchfield (*n* = 2)	8:l,v:1,2	2
Livingstone (*n* = 5)	6,7,14:d:l,w	5
London (*n* = 2)	3,{10}{15}:l,v:1,6	2
Manhattan (*n* = 2)	6,8:d:1,5	2
Mbandaka (*n* = 2)	6,7,14:z_10_:e,n,z_15_	2
Meleagridis (*n* = 1)	3,{10}{15}{15,34}:e,h:l,w	1
Menston (*n* = 1)	6,7:g,s,[t]:[1,6]	1
Minnesota (*n* = 1)	21:b:e,n,x	1
Mississippi (*n* = 1)	1,13,23:b:1,5	1
Montevideo (*n* = 8)	6,7,14:g,m,[p],s:[1,2,7]	8
Muenchen (*n* = 5)	6,8:d:1,2	5
Muenster (*n* = 1)	3,{10}{15}{15,34}:e,h:1,5	1
Newington (*n* = 2)	3,{10}{15}{15,34}:e,h:1,6	2
Newport (*n* = 3)	8:e,h:1,2	3
Ohio (*n* = 4)	6,7,14:b:l,w	4
Oranienburg (*n* = 2)	6,7,14:m,t:[z_57_]	2
Panama (*n* = 3)	1,9,12:l,v:1,5	3
Paratyphi B (*n* = 2)	1,4,[5],12:b:1,2	2
Poona (*n* = 2)	1,13,22:z:1,6	2
Reading (*n* = 3)	1,4,[5],12:e,h:1,5	3
Rissen (*n* = 3)	6,7,14:g,f:-	3
Saintpaul (*n* = 3)	1,4,[5],12:e,h:1,2	3
Schwarzengrund (*n* = 3)	1,4,12,27:d:1,7	3
Senftenberg (*n* = 4)	1,3,19:g,[s],t:-	4
Singapore (*n* = 1)	6,7:k:e,n,x	1
Stanley (*n* = 3)	1,4,[5],12,[27]:d:1,2	3
Tennessee (*n* = 2)	6,7,14:z_29_:[1,2,7]	2
Thompson (*n* = 5)	6,7,14:k:1,5	5
Typhi (*n* = 5)	9,12:d:-	5
Typhimurium (*n* = 8)	1,4,[5],12:i:1,2	8
Uganda (*n* = 1)	3,{10}{15}:l,z_13_:1,5	1
Vinohrady (*n* = 1)	28:m,t:[e,n,z_15_]	1
Virchow (*n* = 8)	6,7,14:r:1,2	8
Weltevreden (*n* = 1)	3,{10}{15}:r:z_6_	1

*^1^Serovar confirmed through antisera aggregation.*

*^2^Number of strains amplified through real-time PCR with corresponding primer pair.*

**TABLE 5:** Serotyping results for isolates with unknown serovars.

Strains	Strain designations	Real-time PCR
KVCC^1^-BA1900349	*Salmonella enterica*	I 4,[5],12:i:-
KVCC-BA1700172	*Salmonella enterica*	Enteritidis
KVCC-BA1700171	*Salmonella enterica*	Enteritidis
KVCC-BA1700170	*Salmonella enterica*	Enteritidis
KVCC-BA1700168	*Salmonella enterica*	Enteritidis
KVCC-BA1700169	*Salmonella enterica*	Enteritidis
KVCC-BA0001439	*Salmonella enterica*	Blockley
KVCC-BA0000429	*Salmonella enterica*	Blockley
KVCC-BA1800008	*Salmonella enterica*	Hadar
KVCC-BA0000718	*Salmonella enterica*	Livingstone
KVCC-BA0000704	*Salmonella enterica*	Livingstone
KVCC-BA1800002	*Salmonella enterica*	Mbandaka
KVCC-BA1800001	*Salmonella enterica*	Mbandaka
KVCC-BA1800010	*Salmonella enterica*	Montevideo
KVCC-BA1900334	*Salmonella enterica*	Rissen
KVCC-BA0701417	*Salmonella enterica*	Typhimurium
KVCC-BA0000703	*Salmonella enterica*	Typhimurium
KVCC-BA0000695	*Salmonella enterica*	Typhimurium
KVCC-BA0000692	*Salmonella enterica*	Typhimurium
KVCC-BA0000691	*Salmonella enterica*	Typhimurium
KVCC-BA0000702	*Salmonella enterica*	Typhimurium
KVCC-BA0000699	*Salmonella enterica*	Typhimurium
KVCC-BA0000683	*Salmonella enterica*	Typhimurium
KVCC-BA0000689	*Salmonella enterica*	Typhimurium
KVCC-BA1300166	*Salmonella* species	Enteritidis
KVCC-BA1300261	*Salmonella* species	Infantis
KVCC-BA1300155	*Salmonella* species	Infantis
KVCC-BA1300151	*Salmonella* species	Montevideo
KVCC-BA1300258	*Salmonella* species	Typhimurium
KVCC-BA1600002	*Salmonella* species	Virchow
KVCC-BA1200044	*Salmonella* species	Infantis
KVCC-BA1800599	*Salmonella* species	London
KVCC-BA1600010	*Salmonella* species	Senftenberg

*^1^KVCC, the Korea Veterinary Culture Collection.*

This method not only can clearly distinguish between two serovars presenting similar antigenic formulas, but also alleviates the time and cost required for traditional serotyping method. This method has the limitation of lack of replicates in a sample run of 60 serovars in 96-well plates, but may be necessary for application in the field as it is evaluated in a single plate.

## Conclusion

In this study, novel serovar-specific gene markers were discovered through pangenome analysis of whole-genome sequences. The pangenome analysis could identify gene markers for 60 *Salmonella* serovars present in genomes of target serovars and absent in genomes of other serovars. Furthermore, *in silico* analyses confirmed that some genomes deposited in the public database, such as the NCBI, were incorrectly designated. The real-time PCR method, designed to detect serovar-specific gene markers using a single 96-well plate, successfully detected 222 strains, thus validating the specificity and effectiveness of the assay. Additionally, the traditional serotyping method yielded ambiguous results for strains that share similar antigenic formulas but were accurately identified as one serovar using real-time PCR. These results suggest that the efficient real-time PCR assay developed could be used as a high-throughput diagnostic tool to identify 60 serovars. The real-time PCR method developed in this study is useful in diagnosing *Salmonella* infections and has applications in food safety and human health.

## Data Availability Statement

The original contributions presented in the study are included in the article/Supplementary Material, further inquiries can be directed to the corresponding author/s.

## Author Contributions

S-MY and H-YK contributed to the conception and design of this study. S-MY, EK, SK, and DoK performed the analysis of *in silico* serotyping and pangenome. S-MY, EK, DaK, and H-BK performed the real-time PCR. JB and HY performed the serotyping using antisera agglutination. S-MY and EK prepared a draft manuscript. H-YK reviewed and edited the manuscript. All authors contributed to manuscript revision, read, and approved the submitted version.

## Conflict of Interest

The authors declare that the research was conducted in the absence of any commercial or financial relationships that could be construed as a potential conflict of interest.

## Publisher’s Note

All claims expressed in this article are solely those of the authors and do not necessarily represent those of their affiliated organizations, or those of the publisher, the editors and the reviewers. Any product that may be evaluated in this article, or claim that may be made by its manufacturer, is not guaranteed or endorsed by the publisher.

## Footnotes

1
http://insilico.ehu.es/PCR/
