# Supplementary material for: Rapid Real-Time Polymerase Chain Reaction for Salmonella Serotyping Based on Novel Unique Gene Markers by Pangenome Analysis
Source: Front Microbiol. 2021 Sep 21;12:750379. doi: 10.3389/fmicb.2021.750379 (PMC8491608; doi:10.3389/fmicb.2021.750379)
Supplement: Supplementary file 1 [file Data_Sheet_1.docx]

***Supplementary Materials***

**
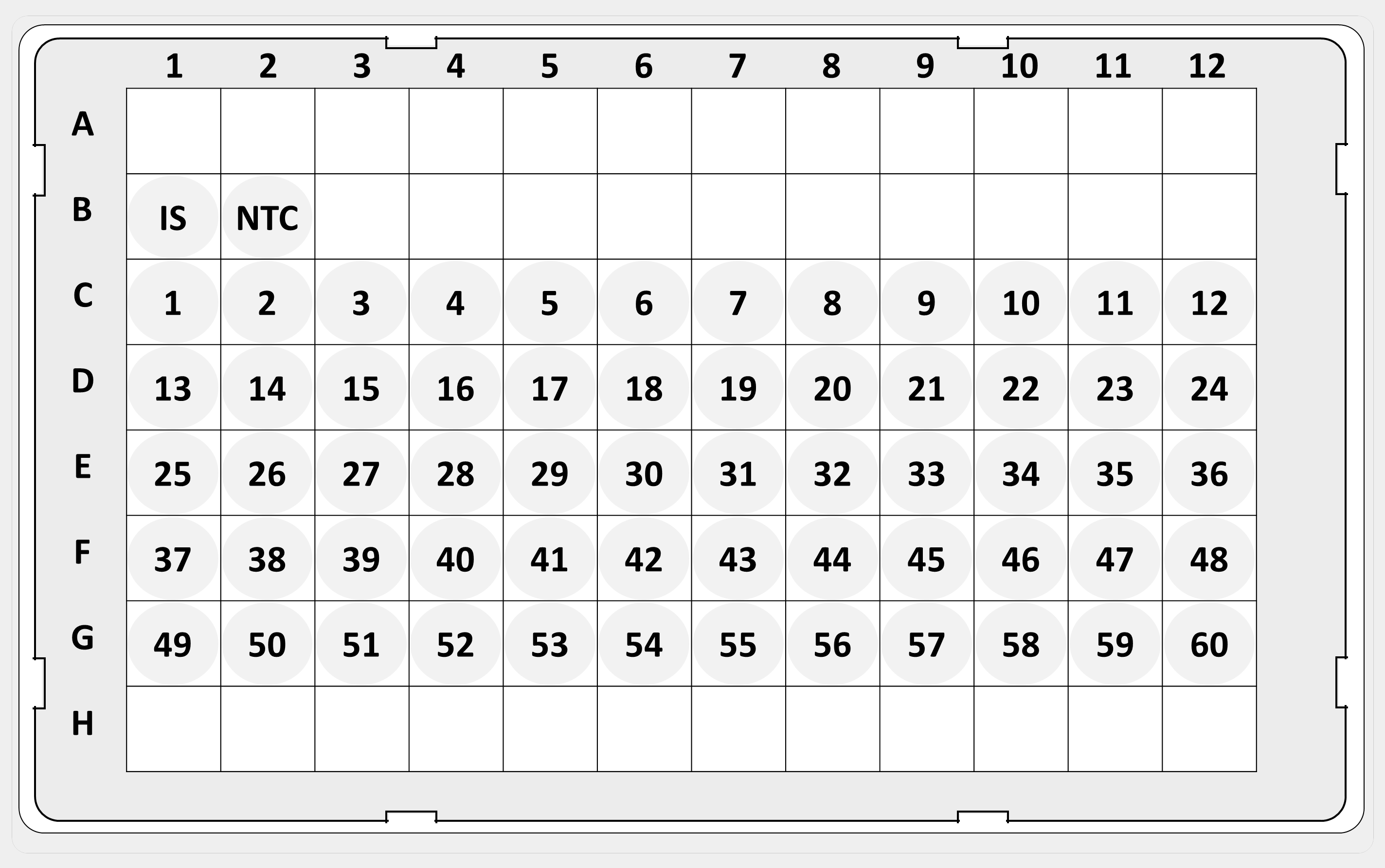
**

**Supplementary Figure 1.** Real-time PCR 96-well plate layout for validation of 60 *Salmonella* serovars. IS, internal standard; NTC, no template control; 1, Aberdeen primer; 2, Agona primer; 3, Albany primer; 4, Anatum primer; 5, Bareilly primer; 6, Berta primer; 7, Blockley primer; 8, Braenderup primer; 9, Brandenburg primer; 10, Cerro primer; 11, Choleraesuis primer; 12, Covallis primer; 13, Derby primer; 14, Dublin primer; 15, Elisabethville primer; 16, Enteritidis primer; 17, Gallinarum primer; 18, Give primer; 19, Hadar primer; 20, Heidelberg primer; 21, I 4,[5],12:i:- primer; 22, Infantis primer; 23, Javiana primer; 24, Kedougou primer; 25, Kentucky primer; 26, Kottbus primer; 27, Litchfield primer; 28, Livingstone primer; 29, London primer; 30, Manhattan primer; 31, Mbandaka primer; 32, Meleagridis primer; 33, Menston primer; 34, Minnesota primer; 35, Mississippi primer; 36, Montevideo primer; 37, Muenchen primer; 38, Muenster primer; 39, Newington primer; 40, Newport primer; 41, Ohio primer; 42, Oranienburg primer; 43, Panama primer; 44, Paratyphi B primer; 45, Poona primer; 46, Reading primer; 47, Rissen primer; 48, Saintpaul primer; 49, Schwarzengrund primer; 50, Senftenberg primer; 51, Singapore primer; 52, Stanley primer; 53, Tennessee primer; 54, Thompson primer; 55, Typhi primer; 56, Typhimurium primer; 57, Uganda primer; 58, Vinohrady primer; 59, Virchow primer; 60, Weltevreden primer.
